# Operational risk assessment of third-party payment platforms: a case study of China

**DOI:** 10.1186/s40854-022-00332-x

**Published:** 2022-03-01

**Authors:** Yinhong Yao, Jianping Li

**Affiliations:** 1 School of Management and Engineering, Capital University of Economics and Business, No. 121 Zhangjialukou Road, Fengtai District, Beijing, 10070 China; 2 University of Chinese Academy of Sciences, Beijing, 100049 China

**Keywords:** Third-party payment (TPP), Operational risk, Loss distribution approach (LDA), Value at risk (VaR), Expected shortfall (ES), G23, G28

## Abstract

Operational risk events have severely impacted the development of third-party payment (TPP) platforms, and have even led to a discussion on the operational risk capital charge settlement by relevant international regulators. However, prior studies have mostly focused on qualitative mechanism analysis, and have rarely examined quantitative risk assessment based on actual operational risk events. Therefore, this study attempts to assess the operational risk on TPP platforms in China by constructing a systematic framework incorporating database construction and risk modeling. First, the operational risk database that covers 202 events between Q1, 2014, and Q2, 2020 is constructed. Then, specific causes are clarified, and the characteristics are analyzed from both the trend and loss severity perspectives. Finally, the piecewise-defined severity distribution based-Loss Distribution Approach (PSD-LDA) with double truncation is utilized to assess the operational risk. Two main conclusions are drawn from the empirical analysis. First, legal risk and external fraud risk are the two main causes of operational risk. Second, the yearly Value at Risk and Expected Shortfall are 724.46 million yuan and 1081.98 million yuan under the 99.9% significance level, respectively. Our results are beneficial for both TPP platform operators and regulators in managing and controlling operational risk.

## Introduction

Third-party payment (TPP) is one of the core forms of the financial technology (Fintech) industry (Chen et al. 2019; Liu et al. 2020; Thakor 2020). As mentioned by Lee and Shin (2018), Yao et al. (2018), and Fan et al. (2020), TPP platforms are independent non-financial institutions that provide payment services connecting bank payments and settlement systems of businesses and commercial banks. The rise of TPP has greatly improved the convenience and applicability of traditional cash and credit card payment transactions (FSB 2017; Xia and Hou 2016; Yao et al. 2018). Since 2013, the TPP industry in China has developed rapidly and has become an important support for driving the retail economy (Greenacre and Akbar 2019; Lenka and Barik 2018; Mawejje and Lakuma 2019). The transaction amounts and deals in Q2, 2020 were 70.22 trillion yuan and 203.5 billion yuan respectively, which have raised by 1219.92% and 2285.23% from the same quarter in 2014. Meanwhile, operational risk events, mainly involving violations of laws and regulations and cyber-attacks, have emerged (Arthur 2017; Xu et al. 2020). Such operational risk events have severely impacted the development of the TPP industry, and have led to a discussion on the operational risk capital settlement by the Committee on Payment and Settlement Systems Board of the International Organization of Securities Commission (CPSS-BIOSC 2013), and the Financial Stability Board (FSB 2017).

The operational risk in traditional financial institutions, including commercial banks and insurance companies, is of great importance for risk identification, measurement, and control (Basel Committee on Banking Supervision [BCBS] 2006; Li et al. 2009; Zhou et al. 2016). Most importantly, a minimum amount of capital is required to cover operational risks (Xu et al. 2019; Zhu et al. 2019). To achieve this, numerous institutions and individuals, such as the Operational Risk Loss Data Exchange Association, British Bankers Association, and Li’s group in China have established an operational risk database for banking and have constructed corresponding risk measurement frameworks (Li et al. 2009; Zhu et al. 2019). However, given the late rise of the Fintech industry and the limited availability of official and privately disclosed data, no institutions or individuals have constructed the operational risk database of TPP platforms, which has created challenges for risk assessment (FSB 2017). Prior studies mostly analyzed the operational risk of TPP platforms qualitatively, such as risk identification and mechanism analysis (FSB 2017; Lee and Shin 2018; Xu et al. 2020). However, there are still many questions that need to be answered when analyzing the operational risk of the emerging TPP industry, especially for the rapidly developing TPP industry in China. For instance, what are the causes and characteristics of operational risk in actual events? How large is the operational risk? It is necessary to fill these gaps by assessing operational risk and analyzing its causes and characteristics for effective management and control in the emerging TPP industry.

Extant studies that analyze the mechanism and identification of operational risk in TPP platforms mainly deal with two aspects. As the core financial form of Fintech, which refers to the technologically enabled innovation in financial services, the TPP platforms have the commonality of survival based on big data, cloud computing, mobile internet, and other emerging technologies (Hedman and Henningsson 2015; FSB 2017). Thus, the main causes of operational risk in the Fintech industry may also exist in the TPP platforms. For the Fintech industry, FSB (2017), Lee and Shin (2018), and Xu et al. (2020) pointed out that the technical risks resulting from cybersecurity and information leakage, operational, credit, and legal risks are the main risk types in Fintech innovation businesses. Morgan (2015) and Gai et al. (2018) discussed the negative impact of cyber security risk on Fintech companies. FSB (2017) suggested that Fintech companies should pay more attention to the detection and prevention of operational risk events to supplement the lack of operational risk capital settlement.

Few studies focus on the operational risk of a specific TPP industry. CPSS-BIOSC (2013) constructed a risk system that includes cyber security, operational, strategic, and legal risks in the TPP industry. Yang et al. (2019) analyzed the threat of insecure in-app payments in the TPP mobile ecosystem. Xia and Hou (2016) and Khalilzadeh et al. (2017) paid more attention to the risk factors that influence consumers’ risk perception of using TPP. Feng and Yuan (2018) assessed operational risk from a consumer perspective. The technical and legal risks in the above studies can also be covered in operational risk. Thus, operational risks in the TPP industry have attracted much attention. However, to the best of our knowledge, no studies have quantitatively assessed the operational risk of TPP platforms.

Recently, numerous operational risk events have occurred in the TPP industry, resulting in severe platform losses. Due to basic technology, TPP platforms may suffer losses from technical factors such as network vulnerabilities, hacking attacks, and speculative cash-outs, and bear the compensation once the user accounts are stolen (Benaroch et al. 2012; FSB 2017). Furthermore, emerging TPP platforms face a more complicated marketing regulatory environment (FSB 2017; Lee and Shin 2018). Under the strict supervision of Fintech businesses in recent years, many TPP platforms have been fined tens of million yuan by regulators for violating payment business regulations, improper anti-money laundering, etc., and have even had their payment licenses canceled. Meanwhile, CPSS-BIOSC (2013) pointed out that TPP platforms should constantly reassess risks to ensure the effectiveness of their risk management system. Therefore, it is necessary to analyze the causes of the operational risk of TPP platforms and assess their risk value.

This study attempts to assess the operational risk of TPP platforms in China by constructing a systematic framework incorporating database construction and risk modeling. Specifically, the operational risk database of TPP platforms is first constructed by collecting loss data from websites based on risk mechanism analysis. The database includes 202 operational risk events between Q1, 2014, and Q2, 2020. Then, the event occurrence causes, frequency, and loss characteristics are analyzed in detail. Finally, according to the heavy tail and truncation characteristics of the loss distribution, the piecewise-defined severity distribution based-Loss Distribution Approach (PSD-LDA) with a doubly-truncated Lognormal distribution and Generalized Pareto Distribution (GPD) is utilized to assess the operational risk. The quarterly operational risks in five significant levels are transformed to a general yearly risk value to be suitable for setting operational risk capital. Additionally, robustness tests based on three simulation types and backtesting tests are conducted to validate the reliability of the database and results.

This study contributes to the existing literature as follows. First, it is a unique study because it comprehensively analyzes the operational risk of the emerging TPP industry in China using actual data. Second, a systematic framework incorporating database construction and risk modeling is constructed and applied to analyze the causes and characteristics of the operational risk and assess the risk value. It is noteworthy that an operational risk database consisting of 202 risk events is first constructed for the emerging TPP industry. Third, PSD-LDA with double-truncated distributions and GPD under four types of simulations is used to assess the operational risk. Our study fills the gap of insufficient quantitative analysis of the operational risk in TPP platforms and clarifies the causes and characteristics based on the collected real data. These results will be beneficial for both platform operators and regulators to understand and control risk occurrences and set up operational risk capital to better supervise the TPP industry.

The remainder of this paper is organized as follows. “Method” section introduces the PSD-LDA framework. “Database construction and characteristic analysis” section constructs an operational risk database and analyzes the loss characteristics. “Empirical results” section empirically calculates the operational risk. “Conclusions and discussions” section presents the conclusions and discussions.

## Method

Considering the similarity of payment businesses of TPP platforms and the payment and settlement line of commercial banks, we mainly draw on the existing mature operational risk measurement framework of traditional financial institutions. Selecting a reliable risk measurement method is very important for precisely assessing the operational risk (Han et al. 2015; Xu et al. 2019). In previous studies, LDA has been widely used as a risk method for assessing operational risk (Wang et al. 2012; Zhou et al. 2016; Zhu et al. 2019). Feng et al. (2012) indicated that LDA is the most accurate model, as it uses the exact distributions of frequency and severity of financial institutions’ loss data. Thus, we apply the LDA model to assess the operational risk of emerging TPP platforms in China. Specifically, the operational risk, value at risk (VaR), and expected shortfall (ES) can be obtained through the aggregated loss distribution simulated by compounding the frequency and severity distributions over a one-year time horizon via convolutions (Chapelle et al. 2008; Li et al. 2009).

**Fig. 1:**
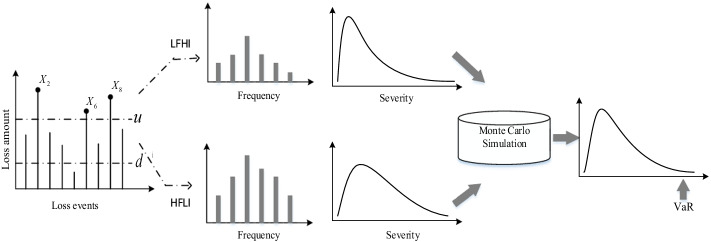
The framework of PSD-LDA with doubly-truncated distribution and GPD

LDA has evolved in many ways to ensure suitability with loss data having different characteristics, such as fat-tail and data truncation. For instance, Jiménez-Rodríguez et al. (2011) applied LDA to assess the operational risk of banks based on left-truncated data. Li et al. (2009) and Wang et al. (2012) proposed a PSD-LDA that combines a parameter form for ordinary losses, and a GPD for extreme losses, and then estimated the operational risk by LDA using a Monte Carlo simulation. Chen (2019) combined the doubly-truncated distributions and the GPD to fit the severity distribution under the LDA framework. In this study, because the operational risk loss data of TPP platforms is left-truncated, the loss distribution with high-frequency low-impact (HFLI) and low-frequency high-impact (LFHI) characteristics cannot be fitted by one distribution function well. These characteristics are analyzed in “Database construction and characteristic analysis” section in detail. Therefore, the PSD-LDA with a doubly-truncated distribution and GPD is utilized to assess the operational risk of China’s TPP platforms. This framework is presented in Fig. 1. Specifically, the six main procedures for estimating VaR and ES are as follows:

### Confirming two thresholds

In PSD-LDA with a doubly truncated distribution and GPD framework, the left truncated point and the threshold that distinguishes large losses from ordinary losses should first be determined. Specifically, the left truncated threshold $d$ is determined by the analysis of operational risk event sources and loss characteristics. The choice of threshold $u$, which is the point for dividing all the losses into HFLI and LFHI parts, and the general difficulty of the Peak Over Threshold (POT) model (Zhu et al. 2019) are critical aspects. Choosing a threshold that is too high leads to very few excesses, and thus a high variance for model estimation, while a low value induces an approximation bias (Han et al. 2015; Li et al. 2009). Thus, two popular methods, the mean excess plot and Hill plot are used to determine the threshold. For the mean excess method, assume there are $n$ loss samples, and let $X_{\left( 1 \right)} > X_{\left( 2 \right)} > \cdots > X_{\left( n \right)}$, then the mean excess function can be expressed as:

1 $$ e\left( u \right) = \frac{{\sum\nolimits_{i = k}^{n} {\left( {X_{i} - u} \right)} }}{n - k - 1},\quad k = \min \left\{ {i|X_{i} > \mu } \right\}. $$

The mean excess plot is a curve consisting of the point $\left( {u,e\left( u \right)} \right)$. The value for $u$ can be chosen as the threshold at which the curve becomes linear (Zhu et al. 2019). For the Hill method, the Hill estimator using the $k$ order statistics is given by:

2 $$ H_{k,n} = \frac{1}{k}\sum\limits_{i = 1}^{k} {\ln \left( {\frac{X\left( i \right)}{{X\left( {k + 1} \right)}}} \right)} . $$

The Hill plot is a plot of the points $\left( {k,H_{k,n} } \right)$. The threshold $u$ is determined at $X_{k}$ with the plot becoming stable from $k$ (Chen 2019). Therefore, the operational losses between $d$ and $u$ are HFLI with truncated samples, and those above $u$ are LFHI losses.

### Fitting the frequency distribution

Fitting the frequency distributions of HFLI and LFHI losses separately is a precondition for simulating operational risk. In this study, loss frequency refers to the number of loss events that occurred quarterly on TPP platforms. The frequency is often modeled by either a homogeneous Poisson distribution (PD) or a negative binomial distribution (NBD; Zhu et al. 2019). The Kolmogorov–Smirnov (KS) goodness-of-fit test, which is widely used to test whether a theoretical distribution is suitable for the empirical data, is used to select the best fitted one (Carrillo-Menézdez and Suárez 2012; Li et al. 2009; Zhu et al. 2019). Specifically, the probability function of the PD is:

3 $$ P\left( {X = k} \right) = \frac{{\lambda^{k} }}{k!}e^{ - \lambda } ,\quad k = 0,1, \ldots . $$

The expected $\lambda$ is estimated by averaging the number of loss events, and $k$ denotes the number that occurred quarterly in this study. The probability function of the NBD is:

4 $$ P\left\{ {X = k} \right\} = \left( {\begin{array}{*{20}c} {k - 1} \\ {r - 1} \\ \end{array} } \right)p^{r} q^{k - r} ,\quad k = r,r + 1, \ldots . $$

where $p$ denotes the probability of occurrence of the $r$th event.

### Fitting the severity distribution

The severity distribution specifies the size of the individual operational risk losses (Li et al. 2009). Because the variations in severity distribution have a significant effect on operational risk assessment, it is important to choose a reliable distribution in the LDA framework (Chen et al. 2019; Zhu et al. 2019). In this study, the collected operational loss data of TPP platforms are left truncated and cannot be properly fitted by only one distribution function. Thus, we use the doubly truncated severity distribution and GPD to fit the HFLI and LFHI losses separately. Specifically, doubly-truncated Lognormal, Gamma, and Weibull distributions are employed to model the severity losses of the HFLI part. For the doubly-truncated distribution, $F\left( {x;\theta } \right)$ is the cumulative distribution function (cdf) of operational risk loss, $f\left( {x;\theta } \right)$ is the corresponding probability density function (pdf), and $\theta$ is a parameter that needs to be estimated (Chen 2019). The conditional cdf and pdf of the loss data between the left truncated point $d$ and threshold $u$ is expressed as:

5 $$ F^{*} \left( {X \le x;\theta |d \le x \le u} \right) = \frac{{F\left( {x;\theta } \right) - F\left( {d;\theta } \right)}}{{F\left( {u;\theta } \right) - F\left( {d;\theta } \right)}}, $$

6 $$ f^{*} \left( {X \le x;\theta |d \le x \le u} \right) = \frac{{f\left( {x;\theta } \right)}}{{F\left( {u;\theta } \right) - F\left( {d;\theta } \right)}}. $$

Therefore, three utilized distribution functions $f^{*} \left( {x;\theta } \right)$ are shown as follows,

1. Doubly-truncated Lognormal distribution
  7 $$ f^{*} \left( {X \le x|d \le x \le u} \right) = \frac{{\exp \left[ { - \left( {\ln x - \mu } \right)^{2} /\left( {2\sigma^{2} } \right)} \right]}}{{\sqrt {2\pi } \sigma x\left[ {\Phi \left( {\frac{\ln u - \mu }{\sigma }} \right) - \Phi \left( {\frac{\ln d - \mu }{\sigma }} \right)} \right]}}. $$
2. Doubly-truncated Gamma distribution
  8 $$ f^{*} \left( {X \le x|d \le x \le u} \right) = \frac{{\beta^{\alpha } x^{\alpha - 1} e^{ - \beta x} }}{{\left[ {\Gamma \left( {\alpha ,\beta u} \right) - \Gamma \left( {\alpha ,\beta d} \right)} \right]\Gamma \left( \alpha \right)}}. $$
3. Doubly-truncated Weibull distribution
  9 $$ f^{*} \left( {X \le x|d \le x \le u} \right) = \frac{k}{\lambda }\left( {\frac{x}{\lambda }} \right)^{k - 1} \exp \left\{ { - \left( {\frac{x}{\lambda }} \right)^{k} } \right\}\frac{1}{{\exp \left\{ { - \left( {\frac{d}{\lambda }} \right)^{k} } \right\} - \exp \left\{ { - \left( {\frac{u}{\lambda }} \right)^{k} } \right\}}}. $$

In the above three functions, $\mu$ and $\sigma$ are the mean and standard deviation of $\ln \left( {loss} \right)$. $\alpha$ and $\beta$ are the shape and inverse scale parameters of the fitted Gamma distribution. $k$ and $\lambda$ are the shape and scale parameters of the fitted Weibull distribution.

For LFHI losses, the GPD, which is the main distribution model for random variables above the threshold $u$ (Li et al. 2009), is chosen as the severity distribution function. Its cdf can be expressed as:

10 $$ F\left( {x;\xi ,\beta ,u} \right) = 1 - \left( {1 + \xi \frac{x - u}{\beta }} \right)^{ - 1/\xi } ,\quad x \ge u,1 + \xi \left( {x - u} \right)/\beta > 0, $$

where $\xi$ and $\beta$ are the shape and scale parameters, respectively. These two parameters can be estimated using the real data of excess losses, and a larger $\xi$ means a heavier tail.

### Total loss distribution aggregated with Monte Carlo simulation

After determining two thresholds and fitting the frequency and severity distributions, the generation of the total loss distribution is an important step for calculating the operational risk. We denote $L$ as the summarization of individual operational risk event loss $X_{i} ,i = 1,2, \ldots ,N$ occurring in a quarter, $L = \sum\nolimits_{i = 1}^{N} {X_{i} }$. The total loss distribution is convolved with the loss frequency and severity distribution, and there is no analytical formula. Thus, the Monte Carlo simulation method is mostly utilized. The four main steps are as follows.

- Step 1: Simulating quarterly HFLI losses
  (1.1) Fit the frequency distribution $F_{1}$ of HFLI losses and randomly generate the frequency number $N_{1}$ of HFLI events.
  (1.2) Fit the severity distribution $S_{1}$ of HFLI losses and randomly generate $x_{1} ,x_{2} , \ldots ,x_{{N_{1} }}$ losses from $S_{1}$.
  (1.3) Calculate the total quarterly loss of HFLI events by summating individual losses, that is, $L_{hl} = \sum\nolimits_{i = 1}^{{N_{1} }} {x_{i} }$.

- Step 2: Simulating quarterly LFHI losses
  (2.1) Fit the frequency distribution $F_{2}$ of LFHI losses and randomly generate the frequency number $N_{2}$ of LFHI events.
  (2.2) Fit the severity distribution $S_{2}$ of LFHI losses and randomly generate $x_{1} ,x_{2} , \ldots ,x_{{N_{2} }}$ losses from $S_{2}$.
  (2.3) Calculate the total quarterly loss of LFHI events by summating individual losses, that is, $L_{lh} = \sum\nolimits_{i = 1}^{{N_{2} }} {x_{i} }$.

- Step 3: Calculate the total quarterly loss $L = L_{hl} + L_{lh}$.

- Step 4: Repeat Steps 1 to 3 to derive $N$ simulated aggregated losses $L_{1} ,L_{2} , \ldots ,L_{N}$, and obtain the quarterly total loss distribution.

In step 4, more simulations indicate a more accurate aggregation of the loss distribution and a longer simulation time. Following Zhu et al. (2019), the simulation number was set to 100,000 to better balance accuracy and time.

### Quarterly VaR and ES calculation

VaR and ES are two popular methods used in operational risk assessments (Han et al. 2015; Wang et al. 2012; Zhu et al. 2020). VaR is defined as the smallest number $l$, such that the probability of loss $L$ exceeding $l$ is not larger than $\left( {1 - \alpha } \right)$ at a specific confidence level $\alpha \in \left( {0,1} \right)$ for a one-quarter holding period.

11 $$ VaR = \inf \left\{ {l:P\left( {L \le l} \right) \le \left( {1 - \alpha } \right)} \right\}. $$

Assuming that the total loss follows a distribution $F$, VaR can be expressed as the quantile of the distribution, that is:

12 $$ VaR = F^{ - 1} \left( \alpha \right). $$

Unlike VaR, which only captures one point of the distribution, and lacks subadditivity and convexity, ES captures tail events better and is more prudent (Yao et al. 2021). It is defined as the mean loss exceeding VaR:

13 $$ ES = E\left( {L_{i} |L_{i} > VaR} \right). $$

In this study, we use both VaR and ES to calculate operational risks under 90%, 95%, 99%, 99.9%, and 99.97% confidence levels to satisfy different supervision requirements.

### Yearly operational risk transformation

Operational risk capital is usually set to cover yearly risk exposure with a confidence level (Xu et al. 2019). According to the principle of time aggregation, the risk is usually adjusted by taking the square root of time $T$ as the multiplier (Daníelsson and Zigrand 2006; Zhu et al. 2021). Thus, the yearly operational risk $VaR_{y,\alpha }$ and $ES_{y,\alpha }$ can be calculated by multiplying the quarterly risk value $VaR_{q,\alpha }$ and $ES_{q,\alpha }$ with $\sqrt 4$:

14 $$ VaR_{y,\alpha } = \sqrt 4 VaR_{q,\alpha } , $$

15 $$ ES_{y,\alpha } = \sqrt 4 ES_{q,\alpha } . $$

## Database construction and characteristic analysis

Relying on emerging technologies, TPP platforms are highly vulnerable to operational risks such as internal personnel operating errors, imperfect technologies, external network attacks, and violation of laws and regulations (CPSS-BIOSC 2013; FSB 2017). However, to the best of our knowledge, no official operational risk identification, assessment, and supervision system has been constructed worldwide, and relevant data collection work has not received much attention (Feng and Yuan 2018; Liu et al. 2020; Mawejje and Lakuma 2019). According to the operational risk research system in banking (BCBS 2006; Li et al. 2009; Xu et al. 2019; Zhou et al. 2016; Zhu et al. 2019), operational risk is defined as the risk of loss in TPP platforms resulting from inadequate or failed internal processes, people, information technology systems, and external events. This definition includes legal risk but excludes strategic and reputational risks. Based on the basic mechanism analysis, we first construct the operational risk database of TPP platforms by collecting loss data from websites. Then, the causes and loss characteristics of operational risk events are analyzed.

### Database construction

The construction of the operational risk database of TPP platforms mainly includes five steps: determining the target platforms, determining the time interval, determining keywords and retrieving from the Internet, recording event features according to the real operational loss events, and checking this information repeatedly. First, the TPP platforms whose third-party payment licenses have been publicly issued by the PBC are chosen as research objects, which includes 237 current payment platforms, along with 34 platforms whose licenses have been canceled, which comes to a total of 271. Then, we collect as much external loss event data as possible. The time interval ranged from Q1, 2014 to Q2, 2020, with a total of 26 quarters. Generally, the operational risk of the banking industry is measured in annual units. However, because the overall establishment time of the TPP is relatively short, quarterly loss events are used and then transformed to yearly risk values. After determining the research object and time interval, we select “TPP platform name”, “defraud”, “virus”, “attack”, “Hacker”, “fraudulent”, “loss”, and “fine” as the keywords according to the definition and then search on the website, to determine the operational risk events. The main websites are PBC, Hexun, and Sohu. Referring to the operational risk database of banking, we analyzed the characteristics of TPP operational risk events and recorded 12 features—the event description, occurrence date, settlement date, loss amount, TPP platforms, event type, specific loss causes, location, and data source. In this study, we mainly analyze the characteristics of the settlement date, loss amount, and specific loss causes.

After repeated checking, we collected 476 operational risk loss events. The losses range from 0.0001 yuan to 65.89 million yuan. However, there is a consensus that the operational risk losses collected from public media may be biased, which makes the number of small losses look lesser than the actual data (Chen 2019; Jiménez-Rodríguez et al. 2011; Shevchenko and Temnov 2009). The unwanted biases affect the accuracy of the operational capital charge (Jiménez-Rodríguez et al. 2011). A higher threshold can better avoid data biases and increase the statistical significance; hence, we assume a relatively large value at 0.1 million yuan as the data collecting threshold, that is, the left-truncated point. Then, the remaining 202 loss data points that are larger than 0.1 million yuan are utilized to assess the operational risk. The operational risk assessment of TPP platforms is based on the following three hypotheses:

1. The operational risk events reported by the major websites are real and the loss value is correct, and are not rumors or the authors’ inference.
2. The operational risk events with a loss value larger than 0.1 million yuan are fully reported and collected in the constructed database.
3. The occurrence frequency and loss severity are uncorrelated.

### Causes of operational risk

The constructed operational risk database of the TPP platforms contains 202 operational risk events. Among them, 192 events, accounting for 95.05%, are losses due to fines by the regulator for violating laws and regulations. A total of 10 cases, accounting for the remaining 4.95%, are caused by users’ compensation losses and speculative behavior, website vulnerabilities, and hacker attacks. According to the banking operational risk research framework, we classify the above two types of events as legal risk and external fraud risk. Although the collected external fraud risks account for a small percentage, they can result in great losses to the platform. For example, in January 2018, 10.81 million yuan was stolen by Liu and others from the AllScore Payment Service Co., by exploiting the vulnerabilities of the bank and payment platform. Additionally, the small number of such collected events does not necessarily mean that the actual occurrence of such risky events is few. The collection amount has a significant relationship with whether the platforms disclose such risks. Therefore, the operational risk assessment framework still includes a small number of external fraud risk events.

Legal risk, which is closely related to external factors, such as stricter supervision and internal factors such as poor operation and management, accounts for the largest losses of TPP platforms. Regulatory institutions mainly include the PBC and the State Administration of Foreign Exchange (SAFE). A punishment event includes many specific regulations. For example, the Tenpay platform was fined 1.49 million yuan by the Shenzhen sub-branch of PBC on August 30, 2019, for violating payment and settlement management, and financial consumer rights protection-related regulations. Thus, we split the punishment items into specific single causes for better statistics and obtained a total of 316 punishment causes. For these reasons, different PBC branches or sub-branches have two types of disclosures. One is that TPP platforms are punished by violating the “Measures for the administration of bank card acquiring business”, “Administrative measures for non-financial payment institutions”, “Administrative measures for prepaid card business of payment institutions”, “Non-bank payment institutions online payment service management approach”, and others. This type of disclosure is relatively general and is recorded as a “violation of business management regulations”. In this study, a total of 154 such cases are collected, accounting for 48.73%.

**Fig. 2:**
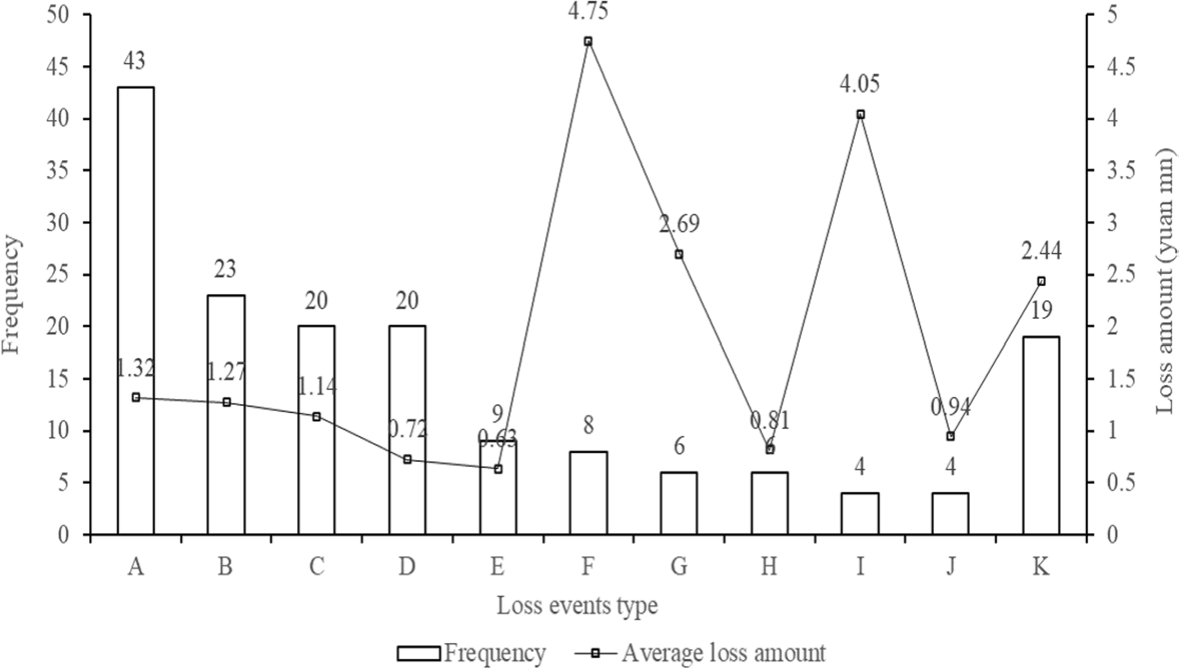
Loss frequency and amount distribution of the second law risk in TPP platforms

The other legal risk type is disclosed in more detail, such as “Failure to submit business data as required”, “Failure to perform customer identification obligations as required”, or “Negligent management of outsourcing business, causing losses to others”. The second type includes 162 events, accounting for 51.27%. We then analyze the second type of legal risk to clarify the main causes of punishment for TPP platforms. The distribution frequency and amount of loss of the second legal risk for TPP platforms are shown in Fig. 2. The loss event frequency decreased from A to K, represented by “Failure to perform customer identification obligations”, “Failure to submit suspicious transaction reports”, “Violation of anti-money laundering regulation”, “Failure to save customer information”, “Failure to manage special engaged merchants”, “Illegal transfer of foreign exchange”, “Risk management measures are not in compliance with regulations”, “Improper management of outsourcing business”, “Improper use of customer reserve fund”, “Does not truly reflect transaction information”, and others.

In these 11 kinds of legal risk types, “Failure to perform customer identification obligation”, “Failure to submit suspicious transaction reports”, “Violation of anti-money laundering regulation”, and “Failure to save customer information” are the top four legal risk types, accounting for 26.54%, 14.20%, 12.35%, and 12.35% respectively. The top three types with the biggest average losses are “Illegal transfer of foreign exchange”, “Improper use of customer reserve fund”, and “Risk management measures are not in compliance with regulations”, which caused losses of 4.75 million yuan, 4.05 million yuan and 2.69 million yuan per loss event, respectively. Other loss events denoted by “K” include “Unauthorized suspension of payment business”, “Do not comprehensively collect system information”, “Illegal place mobile POS”, and so on, causing an average loss of 2.44 million yuan per event. This distribution illustrates that TPP platforms are often punished for violating basic business management regulations, while they are severely damaged by some main events, which indicates the HFLI and LFHI characteristics of operational risk in this emerging TPP industry.

In general, the identified operational risk causes, such as external fraud risk, and the legal risk including “Failure to perform customer identification obligations” and “Improper use of customer reserve fund” are consistent with the analyzed operational risk factors in Xia and Hou (2016), FSB (2017), and Gai et al. (2018). Additionally, we also identified other causes, such as “Failure to submit suspicious transaction reports” and “Failure to manage specially engaged merchants”, which could provide more suggestions for supervising TPP platforms.

### Characteristics of operational risk

#### Trend characteristic

**Fig. 3:**
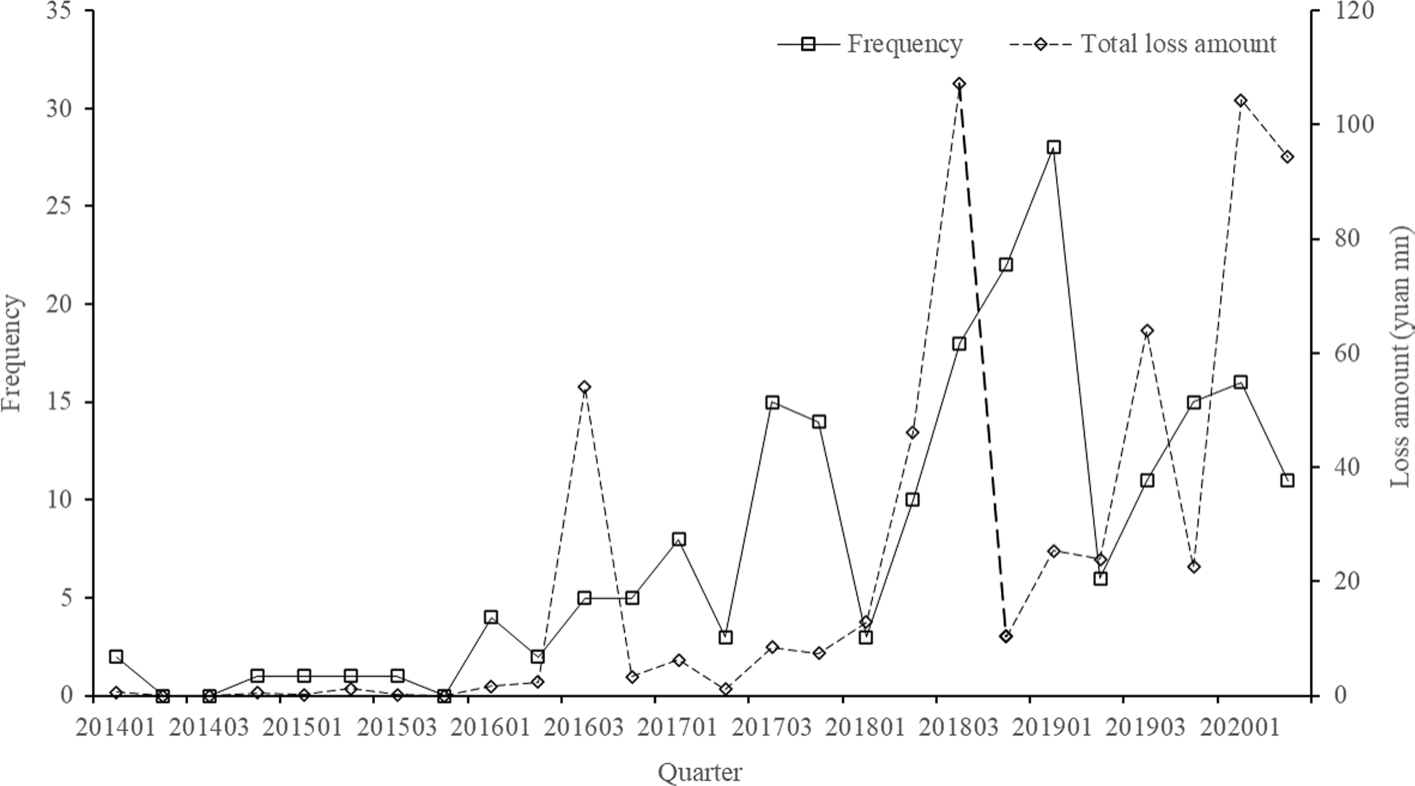
Quarterly loss frequency and amount distribution of the operational in TPP platforms

The quarterly occurrence frequency and loss amount of operational risk events are closely related to the TPP’s development background. Changes in the external regulatory environment and internal management measures can lead to changes in operational risk events. Figure 3 displays the quarterly occurrence frequency and loss amount of operational risk in TPP platforms between Q1, 2014, and Q2, 2020.

Figure 3 shows that the frequency of operational risk events has a trend of first increasing and then fluctuating over time. We speculate that this trend is related to the gradual tightening of supervision since 2015. The “Non-bank payment institutions online payment service management approach” issued by the PBC on December 28, 2015, and implemented on July 1, 2016, indicates that online payment services have basic rules to follow. Thus, the frequency from Q1, 2016 to Q2, 2020 is selected to fit the frequency distribution in the risk assessment framework in this study (Li et al. 2009). A series of laws and regulations were subsequently issued to regulate the development of the TPP industry. Also, the loss amount of operational risk has a trend of first rising, then falling, and finally increasing, and there is no obvious relationship with loss frequency. The characteristic of HFLI and LFHI exist in several quarters. These results indicate the increasingly strict supervision and higher penalties.

#### Loss severity characteristic

**Fig. 4:**
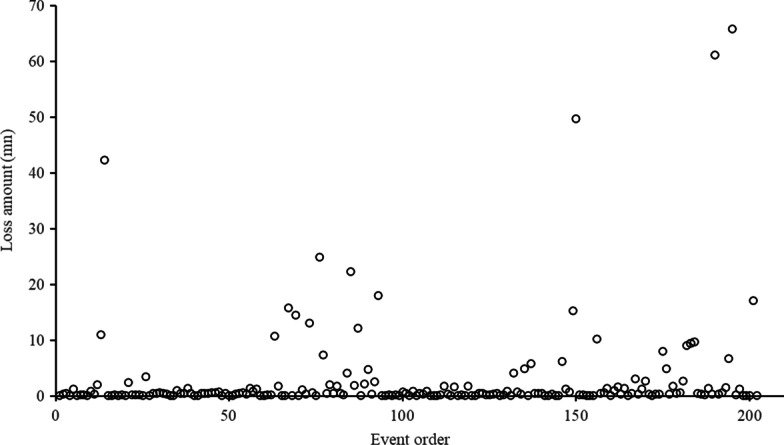
Loss amount distribution of the operational in TPP platforms

The 202 operational risk events are arranged in the order of occurrence. As shown in Fig. 4, it is obvious that the loss severity of individual operational risk events is clustered, that is, most of the losses are concentrated at the bottom with relatively few loss amounts, while few losses far exceed most of the loss. These results are consistent with the two-dimensional HFLI and LFHI characteristics that operational risk events are generally divided into.

Furthermore, the statistical characteristics of operational loss amount are given in Table 1. The minimum loss is 0.1 million yuan, and the largest one is 65.89 million yuan. The difference between these two values is 658.9 times, indicating that the loss data is relatively scattered. The larger standard deviation (denoted as Std) at 8.50 also indicates the larger decentralization. The median value is less than the average, and the skewness is larger than zero, indicating that the loss severity distribution is right-skewed, with more extreme values at the right end of the distribution. The kurtosis is much larger than 3, indicating that the distribution is steeper than the normal distribution and has a sharp peak. The statistical characteristics of loss distribution are similar to the result of the operational risk of banking (Chen 2019; Jiménez-Rodríguez et al. 2011; Li et al. 2009; Wang et al. 2012). This result also indicates that the widely-used LDA in assessing the operational risk of banking can be applied in this study.

**Table 1:** Statistical characteristics of operational loss amount (yuan mn)

Statistics	Minimum	Maximum	Median	Mean	Std	Skewness	Kurtosis
Value	0.1	65.89	0.49	3.01	8.50	5.17	30.10

*Std* standard deviation

## Empirical results

Based on the constructed operational risk database of TPP platforms in China, PSD-LDA with double truncation is applied to assess the operational risk. The threshold determination, parameter estimation of loss frequency and severity distribution, VaR and ES calculation under five significance levels, and backtesting and robustness tests are given in this section.

### Threshold determination

**Fig. 5:**
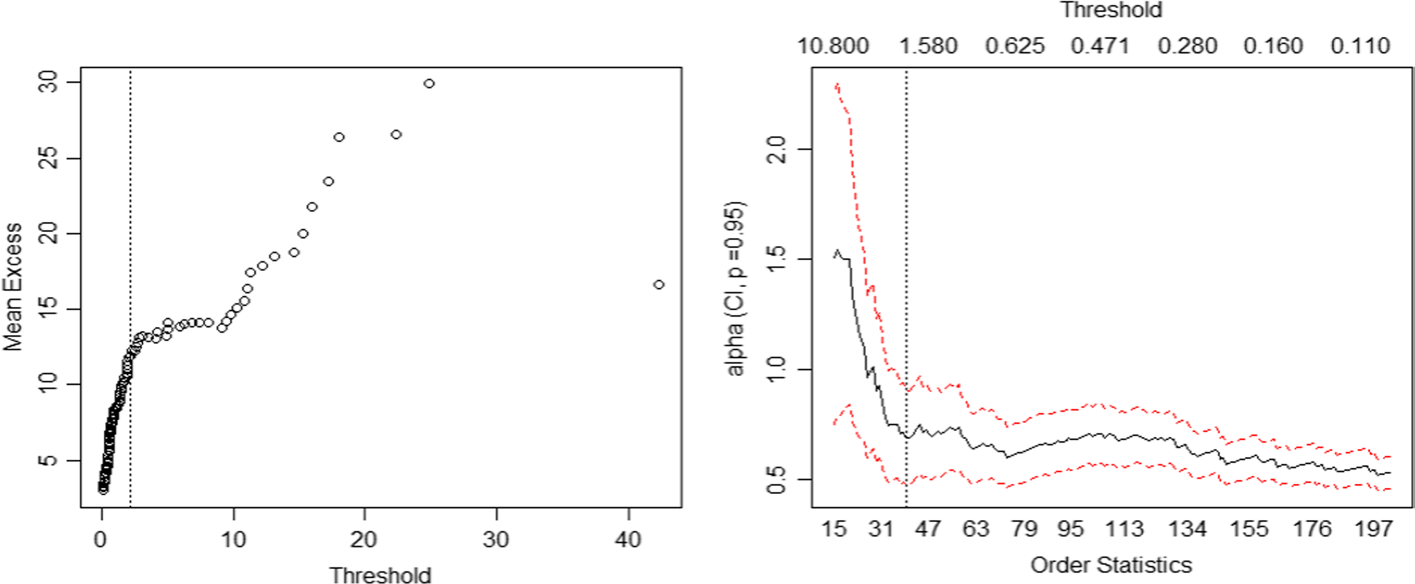
Mean excess plot (left) and Hill plot (right)

Two thresholds used for the PSD-LDA framework are described in this subsection. First, the left truncated point $d$ is determined at 0.1 million yuan based on analysis in previous sections. Then, the threshold $u$ used to divide all the losses into HFLI and LFHI losses is obtained by both the mean excess plot and Hill plot, as shown in Fig. 5.

There is a structural change at 2 million yuan in the mean excess plot, and a smooth upward trend is shown at the vertical dashed line in the Hill plot, which also has a turning point at 2 million yuan. Comprehensively considering these two results, the threshold $u$ for dividing all losses into HFLI and LFHI is determined at 2 million yuan. According to these two thresholds, 38 losses exceeded the threshold $u$ and 164 losses were lower than the threshold, accounting for 18.81% and 81.19% respectively.

### Parameter estimation

The parameters of frequency and severity distributions are calculated using the maximum likelihood estimation method, which is widely used in distribution fitting (Xu et al. 2019; Zhu et al. 2019). First, we choose the best-fitting frequency distributions for the HFLI and LFHI losses. Specifically, the NBD and PD are both utilized to fit the frequency of HFLI, and the latter is also used to fit the frequency of LFHI. The KS test is applied to choose the best one (Li et al. 2009; Zhu et al. 2019). For the KS test, the larger the P-value, the better the fit of the distribution. The significance level is generally set at 5%, which means that we believe the fitted distribution conforms to the theoretical distribution when the P-value is larger than 5%. The estimated parameters and KS test results for the three fitted frequency distributions are listed in Table 2.

**Table 2:** Parameters estimation and KS test results of loss frequency distributions

Loss part	Distribution	Parameter value		D-value	*P* value
HFLI losses	NBD	$r = 2.42$	$p = {0}{\text{.22}}$	0.13	0.90
	PD	$\lambda = 8.78$	–	0.33	0.03
LFHI losses	PD	$\lambda = 2.11$	–	0.21	0.34

The values $r,p$ and $\lambda$ are parameters of NBD and PD respectively.

From Table 2, for HFLI losses, the P-value of the NBD is 0.90, which is significantly larger than 0.05. The *p*-value of the PD with $\lambda = 8.78$, is less than 0.05. Thus, we choose the NBD with the successful number $r = 2.42$, and the successful probability $p = 0.22$ to fit the frequency distribution of the HFLI losses. For the LFHI losses, the fitted PD with $\lambda = 2.11$ passes the KS test with a *p*-value larger than 0.05. For simplicity, the PD is used to fit the frequencies of the LHFI losses.

Then, the best-fitting severity distributions for the HFLI and LFHI losses are also selected. Specifically, the doubly-truncated lognormal distribution, Weibull distribution, and Gamma distribution are all used to model the severity distribution for HFLI losses, and the GPD is used to fit the severity distribution for LFHI losses. Additionally, we also use another popular Anderson–Darling (AD) goodness-of-fit test to choose the best-fitted severity distribution (Carrillo-Menézdez and Suárez 2012). The estimated parameters and two goodness-of-fit test results for the severity distribution fitting are listed in Table 3.

**Table 3:** Parameter estimation and KS test results of loss severity distributions

Loss part	Distribution	Parameter value		KS test	AD test
HFLI losses	Lognormal	$\mu = { - 2}{\text{.31}}$	$\sigma = {2}{\text{.23}}$	0.07 (0.41)	1.01 (0.35)
	Gamma	$\alpha = {0}{\text{.01}}$	$\beta = {0}{\text{.55}}$	0.07 (0.34)	1.22 (0.26)
	Weibull	$k = {0}{\text{.32}}$	$\lambda = {0}{\text{.06}}$	0.07 (0.40)	1.09 (0.31)
LFHI losses	GPD	$\xi = {0}{\text{.35}}$	$\sigma = {7}{\text{.88}}$	0.09 (0.88)	0.41(0.84)

The values $\mu ,\sigma ,\alpha ,\beta ,k,\lambda$ are parameters of three doubly-truncated severity distributions, the values $\xi ,\sigma$ are parameters of GPD; () is the P-value of KS and AD tests.

**Fig. 6:**
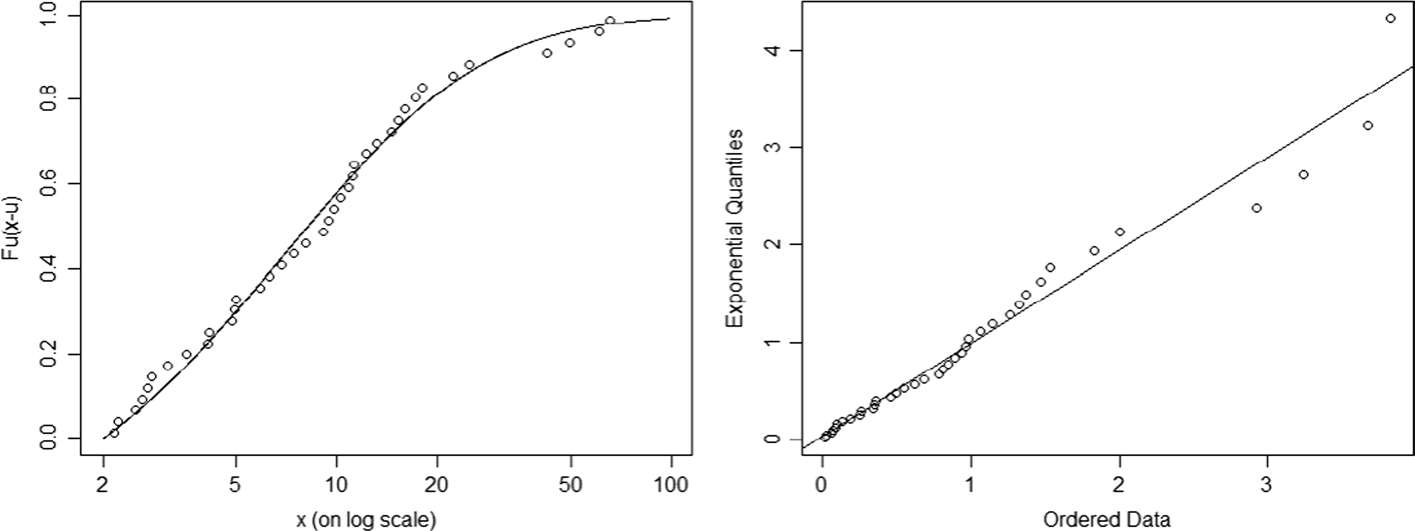
Excess distribution plot (left) and QQ plot of residuals (right)

From Table 3, for HFLI losses, the KS test results reveal that all three doubly-truncated distributions can fit the loss distribution with P-values of 0.41, 0.34, and 0.40, respectively. The doubly-truncated lognormal distribution with the largest P-value is the best fit. For LFHI losses, the GPD can fit the extreme loss data with a P-value significantly larger than 0.05. These results can also be verified by the AD test, which indicates that the fitted doubly truncated Lognormal distribution and GPD are appropriate. Additionally, the excess plot distribution and QQ plot of the residuals are shown in Fig. 6 to verify the effectiveness of the fitted GPD distribution.

From Fig. 6, the excess distribution plot shows that the actual extreme loss data are closely distributed on both sides of the theoretical distribution curve, indicating that the GPD can better fit the extreme loss data of TPP platforms. The QQ plot of residuals that is approximately a straight line across 45° also shows that the distribution fits well. These results also indicate that we have selected an appropriate threshold.

### VaR and ES calculation

As shown in the Method section, once the frequency and severity distributions of HFLI and LFHI losses are determined, the quarterly VaR can be calculated by Monte Carlo simulation, and the yearly VaR can be obtained by multiplying the transformation coefficient. Thus, we obtain the quarterly and yearly VaR at 90%, 95%, 99%, 99.9%, and 99.97% confidence levels, as shown in Table 4. This shows that the VaR value increases as the confidence level increases. For quarterly VaR, it means that the quarterly maximum loss of TPP platforms at five confidence levels are 73.1 million yuan, 97.29 million yuan, 169.95 million yuan, 362.23 million yuan, and 538.95 million yuan, respectively. The calculation of quarterly operational risk can not only help TPP platforms recognize the risk status over one quarter, but also provides a basis for the platform’s operational risk capital settlement in the next stage.

**Table 4:** Quarterly and yearly VaR of China' TPP platforms (yuan mn)

VaR	90%	95%	99%	99.9%	99.97%
Quarterly	73.01	97.29	169.95	362.23	538.95
Yearly	146.04	194.58	339.89	724.46	1077.91

More conventionally, the 99.9% level is used to determine the economic capital requirement for protecting against losses over one year, and the BCBS also recommends 99.9% as a suitable confidence level (Zhu et al. 2019). As the BCBS (2006) mentioned, if the bank can prove that it has taken precautions against expected loss, which is quantified by the mean of the loss distribution, then, the required capital is the VaR at a 99.9% level minus expected losses. As TPP platforms have not yet begun to manage the operational risk, the capital of operational risk should be VaR at the 99.9% confidence level over one year. Therefore, as shown in Table 4, we conclude that the estimated yearly operational risk of TPP platforms in China at the 99.9% confidence level is 724.46 million yuan.

**Fig. 7:**
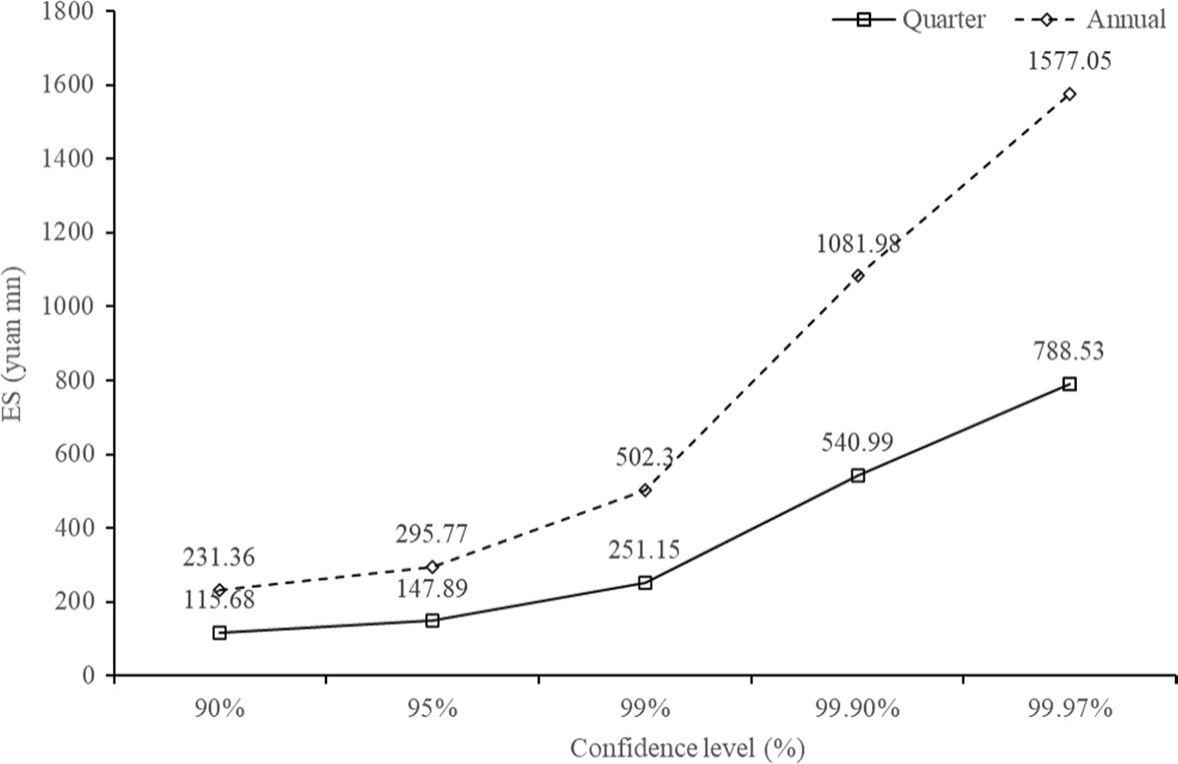
Quarterly and yearly ES of China' TPP platforms (yuan mn)

As ES is a more prudent risk measure than VaR and has also been emphasized by BCBS (2006), we use corresponding quarterly and yearly ES values at five confidence levels. Figure 7 shows that the ES values also increased with the level ranges from 90 to 99.97%, and are larger than the VaR values at the same confidence level. Under the ES criterion, TPP platforms should allocate 540.99 million yuan and 1081.98 million yuan, respectively, to protect against losses over one quarter and one year at the 99.9% level.

### Backtesting and robustness test

To test the accuracy of VaR and ES, the likelihood ratio (LR) proposed by Kupiec (1995) and utilized in the operational risk test in Wang et al. (2012), is applied in this study. Because the scale of the constructed operational risk database is small, we obtain 255 quarterly loss data points that are close to the real number by summarizing any three-month loss for backtesting. This number is determined according to the acceptance region table in Kupiec (1995) and Wang et al. (2012), which provide the acceptance region when the observed number equals 255, 510, and 1000, respectively.

As shown in Table 5, the failure numbers of VaR and ES at 99% and 99.9% levels are 12, 0, 1, and 0 respectively, which nearly conform to the numbers in the acceptance region in Kupiec (1995). According to the LR test at the 0.01 significance level, other than the VaR at the 99% level, the other three risk values all pass through the LR test, which proves that the calculated VaR and ES values are close to the real losses and can be used for operational risk assessment.

**Table 5:** Back-testing results for VaR and ES

Risk value	VaR_99%_	VaR_99.9%_	ES_99%_	ES_99.9%_
Failure number	12	0	1	0
LR	18.63%	5.13%	1.24%	5.13%

The critical values of LR at significance levels 0.01 is 6.63

Additionally, we design three other kinds of simulations, by changing the simulation number, selecting part of the loss data, and using half-annual loss data, to test the stability and sustainability of the constructed database. For the first kind of simulation, we change the simulation number from 100,000 to 1,000,000. Second, we select part of the quarterly loss data that ranges from Q2, 2014 to Q2, 2020. Third, the half-annual loss data, other than the original quarter loss data, are used to fit the frequency and severity distributions. The yearly VaR and ES values and their percentage changes relative to the original results are listed in Table 6. The recalculated VaR and ES are all very close to the original risk value, indicating that the simulation number, loss data volume, and loss data frequency all have less effect on the results. Thus, we infer that the database is stable and sustainable.

**Table 6:** Robustness test for VaR and ES using different simulations

		VaR_99%_	VaR_99.9%_	ES_99%_	ES_99.9%_
Original Value		339.89	724.46	502.30	1081.98
Robustness test	Changing the simulation number	338.52 (− 0.40%)	706.91 (− 2.42%)	503.98 (0.33%)	1108.09 (2.41%)
	Selecting part of the quarterly loss data	335.75 (− 1.22%)	718.54 (− 0.82%)	507.43 (1.02%)	1095.76 (1.27%)
	Using half-annual loss data	351.87 (3.52%)	691.51 (− 4.54%)	494.53 (− 1.55%)	994.25 (− 8.11%)

() is the percentage change against the original value

## Conclusions and discussions

In this study, we attempted to assess the operational risk of TPP platforms in China by constructing a systematic framework incorporating database construction and risk modeling. First, based on the basic mechanism analysis of operational risk events in TPP platforms, an operational risk database of China’s TPP platforms containing 202 events with 12 features ranging from Q1, 2014 to Q2, 2020 was constructed. It is noteworthy that the loss data is left-truncated with a loss value larger than 0.1 million yuan. Then, the specific causes, occurrence trend, frequency, and severity characteristics of individual operational risk loss events were analyzed in detail. Finally, the PSD-LDA model with a doubly-truncated severity distribution and GPD was utilized to assess the operational risk of TPP platforms.

### Conclusions

Two main results were obtained from the empirical analysis. First, the operational risk of the TPP platforms mainly comes from the penalty losses for violating laws and regulations, which are denoted as legal risk, and the external fraud risk caused by users’ compensation losses, speculative behavior, website vulnerabilities, and hacker attacks. Second, quarterly and yearly VaR and ES were calculated under the 90%, 95%, 99%, 99.9%, and 99.97% confidence levels. Yearly VaR values at the five levels are 146.04 million yuan, 194.58 million yuan, 339.89 million yuan, 724.46 million yuan, and 1077.91 million yuan, respectively. With a more prudent ES criterion under 99.9% levels, TPP platforms should prepare 540.99 million yuan and 1081.98 million yuan, respectively, to protect against losses over one quarter and one year.

### Discussions

Compared to prior studies, we obtained consistent results, identifying operational risk causes such as external fraud risk and sections of legal risk. More quantitative results of these causes were given, and the average loss amount and frequency of all causes, the trend of a total loss, and the statistical characteristics of each loss were analyzed in detail. The statistical results of the loss are similar to the characteristics of the operational risk of banking. The main difference is that we assessed the operational risk value, which fills the gap of insufficient quantitative analysis of the operational risk in TPP platforms.

In general, this study has both academic and practical applications. For academic applications, we designed a systematic framework incorporating database construction and risk modeling, and the PSD-LDA model with doubly-truncated severity distributions was used to assess the operational risk. This framework could provide more suggestions for analyzing the operational risk in other emerging industries, such as collecting operational risk event data, clarifying the causes and characteristics of operational risk, and assessing the operational risk value. Our manuscript focuses more on the quantitative analysis of the operational risk in the emerging TPP industry based on real collected data. These results could help platform operators and regulators manage and avoid operational risk events and set up operational risk capital to supervise the TPP industry.

This study has several limitations. The empirical results have not been compared with the operational risk of TPP platforms with those of other countries. Moreover, COVID-19 has affected the operational risk, which has not been analyzed in this study. More risk factors can also be identified with the expansion of the operational risk database. Therefore, this study can be further improved by addressing these limitations based on the expansion of the operational risk database in the future.

## Data Availability

The data for this paper is available upon request.
